# Budd-Chiari syndrome due to right hepatic lobe herniation: CT image findings of two rare clinical conditions

**DOI:** 10.1259/bjrcr.20160133

**Published:** 2017-03-30

**Authors:** Shyamal Saujani, Safi Rahman, Bruce Fox

**Affiliations:** Department of Radiology, Plymouth Hospitals NHS Trust, Plymouth, UK

## Abstract

Hepatic herniation is a rare clinical condition. Most commonly it is associated with congenital diaphragmatic herniation or acquired through blunt diaphragmatic trauma. We present a case of a right hepatic lobe incisional hernia in a 75-year-old female who underwent partial right-sided nephrectomy 52 years previously. Evidence of partial Budd-Chiari syndrome was seen on CT scan that was presumed to be as a result of traction of the herniated liver. As far as we are aware this is the first case of a right-sided hepatic hernia with evidence of partial Budd-Chiari syndrome. The patient was treated conservatively with anticoagulation and analgesia.

## Summary

Hepatic herniation is a rare clinical condition. Most commonly it is associated with congenital diaphragmatic herniation or acquired through blunt diaphragmatic trauma. We present a case of a right hepatic lobe incisional hernia in a 75-year-old female who underwent partial right-sided nephrectomy 52 years previously. Evidence of partial Budd-Chiari syndrome was seen on CT scan that was presumed to be as a result of traction of the herniated liver. As far as we are aware this is the first case of a right-sided hepatic hernia with evidence of partial Budd-Chiari syndrome. The patient was treated conservatively with anticoagulation and analgesia.

## Case presentation

A 75-year-old female presented insidiously with right upper quadrant abdominal tightness and band-like pain. This was associated with a right flank swelling. She had been suffering with progressively worsening abdominal discomfort for many years. There was a 2.5 kg history of weight loss, but no other associated symptoms including ascites or jaundice. Her past medical history included a right partial nephrectomy undertaken in her early 20 s for a renal abscess. The patient also had a history of atrial fibrillation managed with Rivaroxaban.

On examination the patient had a large body habitus and a right-sided subcostal “hockey-stick” scar. Lateral to this scar was a reducible, soft and non-tender hernia with no cough impulse. No hepatomegaly could be felt.

## Investigations

All blood tests (including liver function) were normal. As an investigation of weight loss, a contrast enhanced CT abdomen was performed. A GE HD 64 slice CT scanner was used with the following configuration: *120 kV, 258 mA, slice thickness of 5 mm and spacing of 1.25 mm. *Niopam contrast medium (70 ml) was injected at 3 ml s^–1^ with a scan delay time of 70 s, followed by a 30 ml bolus of 0.9% normal saline at 3 ml s^–1^.

## Findings

**Figure 1.**
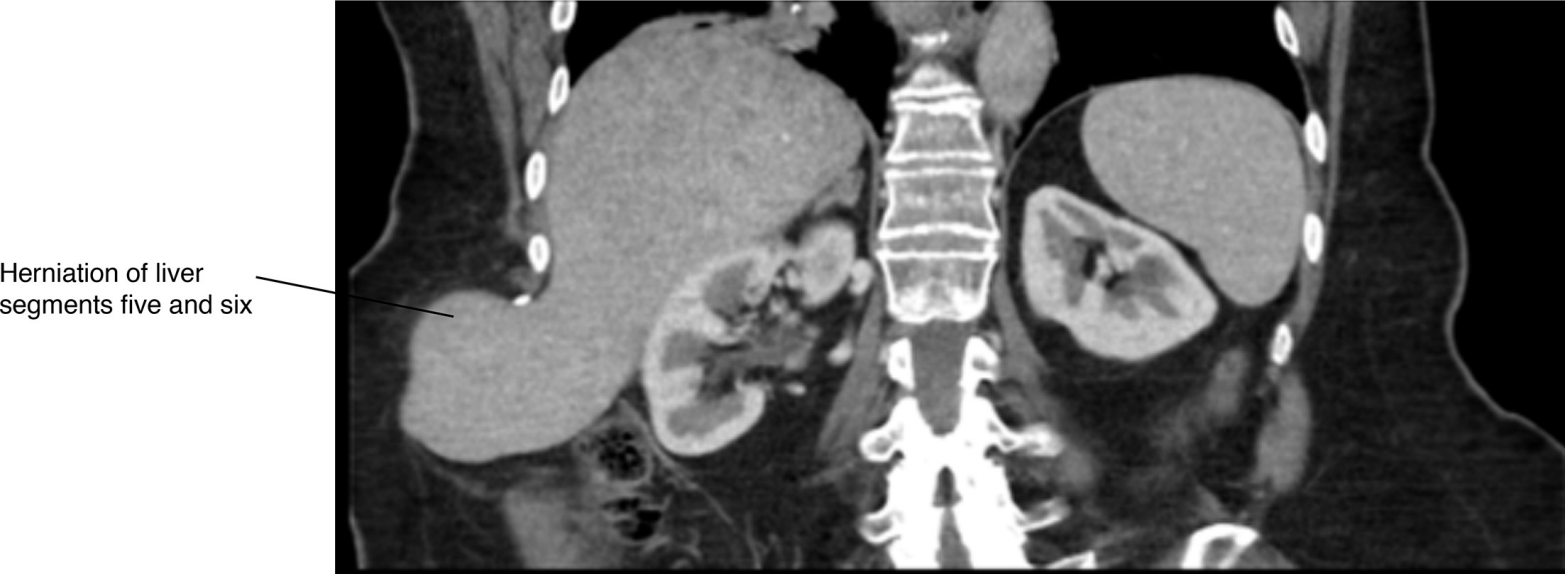
Herniation of liver segments five and six.

**Figure 2.**
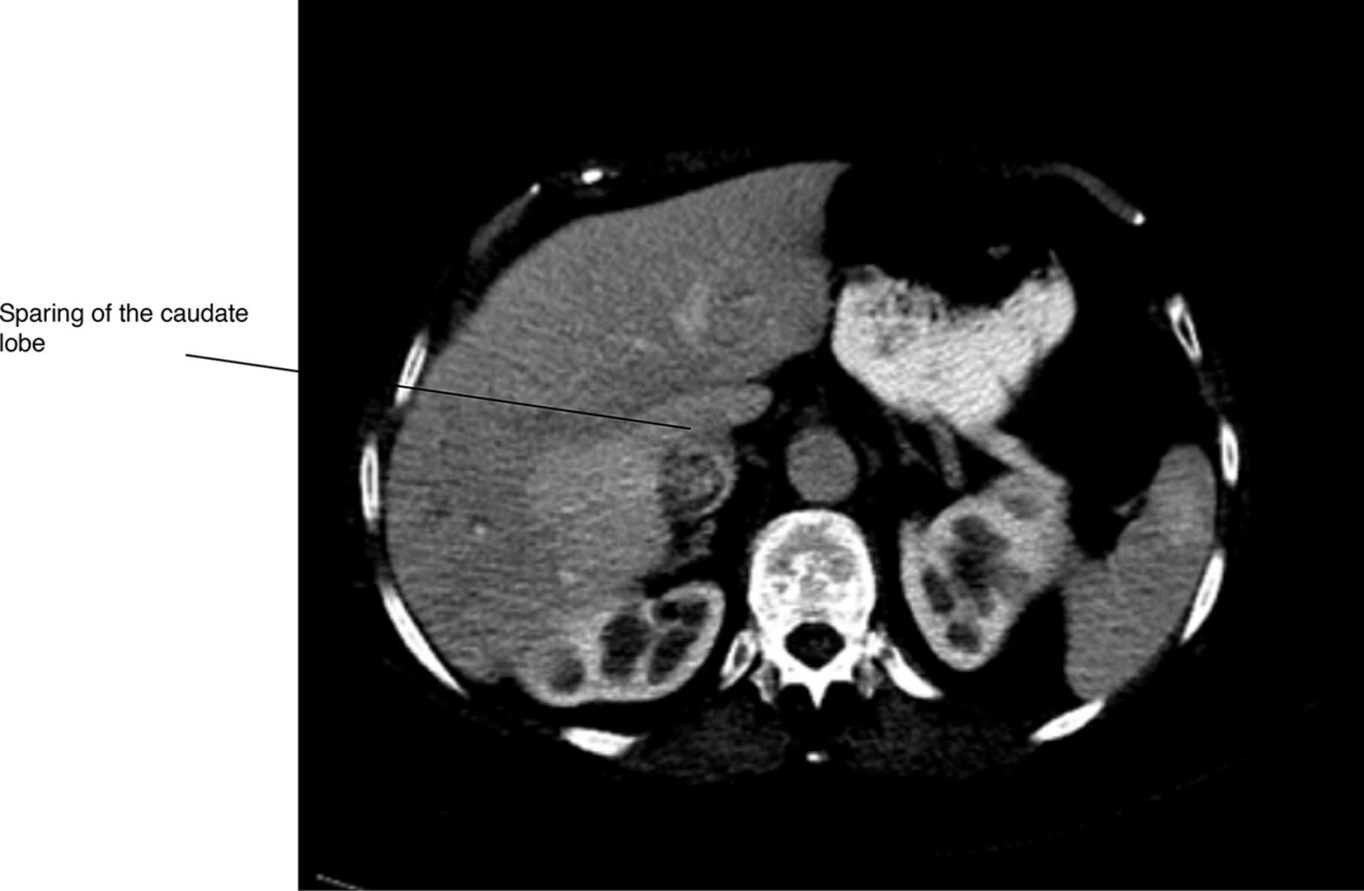
Sparing of the caudate lobe.

**Figure 3.**
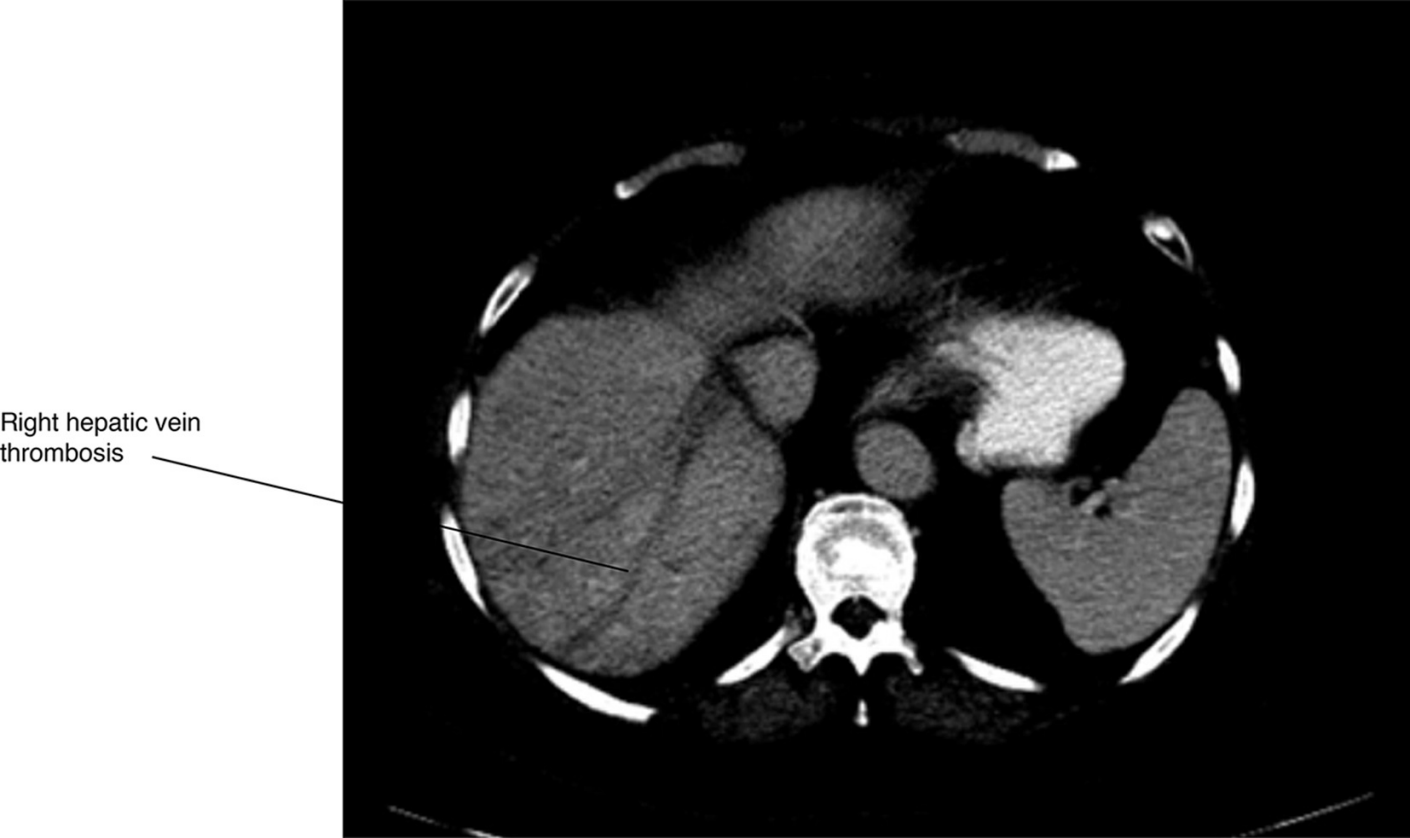
Right hepatic vein thrombosis.

**Figure 4.**
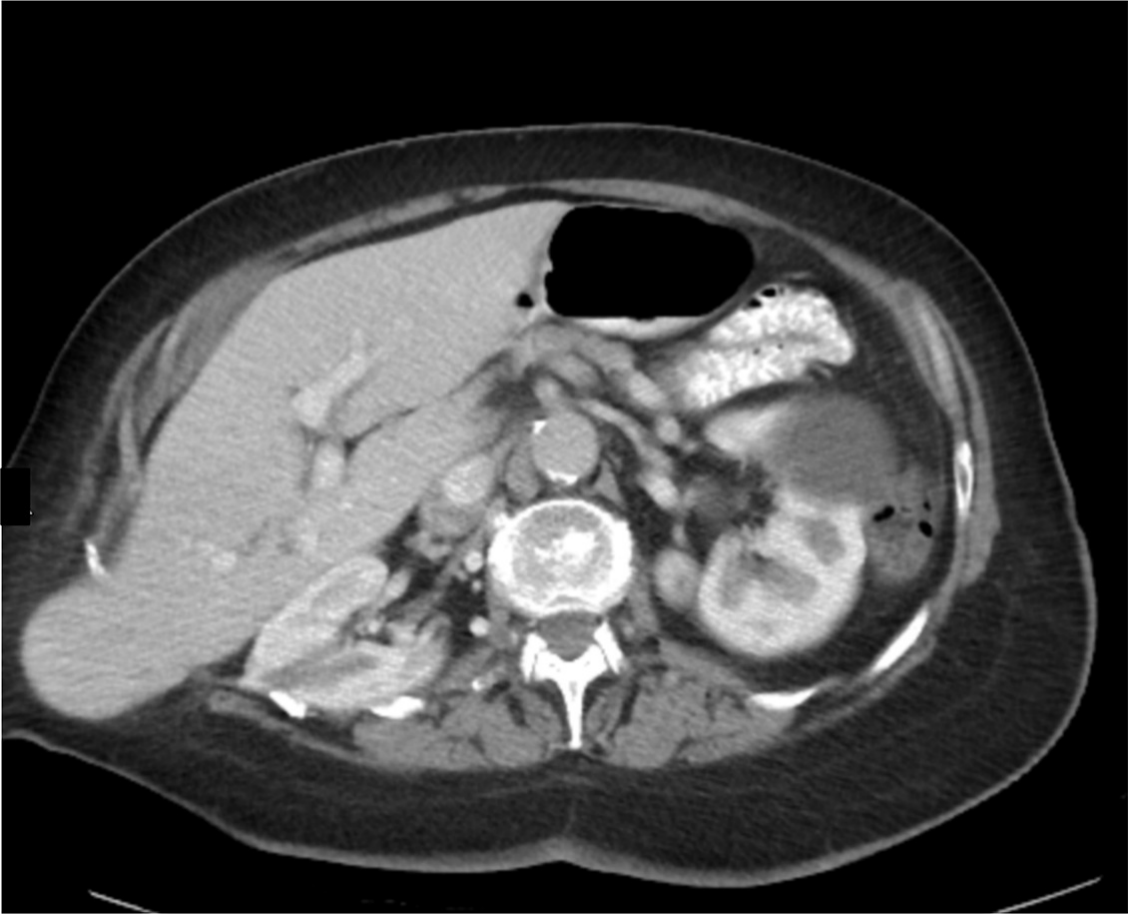
Axial view of CT showing hernia.

The CT scan established the diagnosis of liver herniation of segments 5 and 6 through a right posterolateral hernia corresponding to the site of the partial nephrectomy scar (Figures 1–4). The hernia opening was well above the iliac crest and only partially rounded by latissimus dorsi suggesting this was an incisional hernia and not a Petit’s hernia. There was non-enhancement of the right and middle hepatic veins and venules, with reduced liver parenchymal enhancement within the affected regions. There was increased enhancement of the caudate lobe compared to the left lobe of the liver. These findings were consistent with thrombosis of the right hepatic vein and middle hepatic vein with radiographic evidence of partial, chronic Budd-Chiari syndrome. Owing to a lack of identifiable cause of the hepatic thrombosis based on all blood tests, it was presumed that the aetiology of the Budd-Chiari syndrome was related to traction from the partially herniated liver.

## Treatment

**Figure 5.**
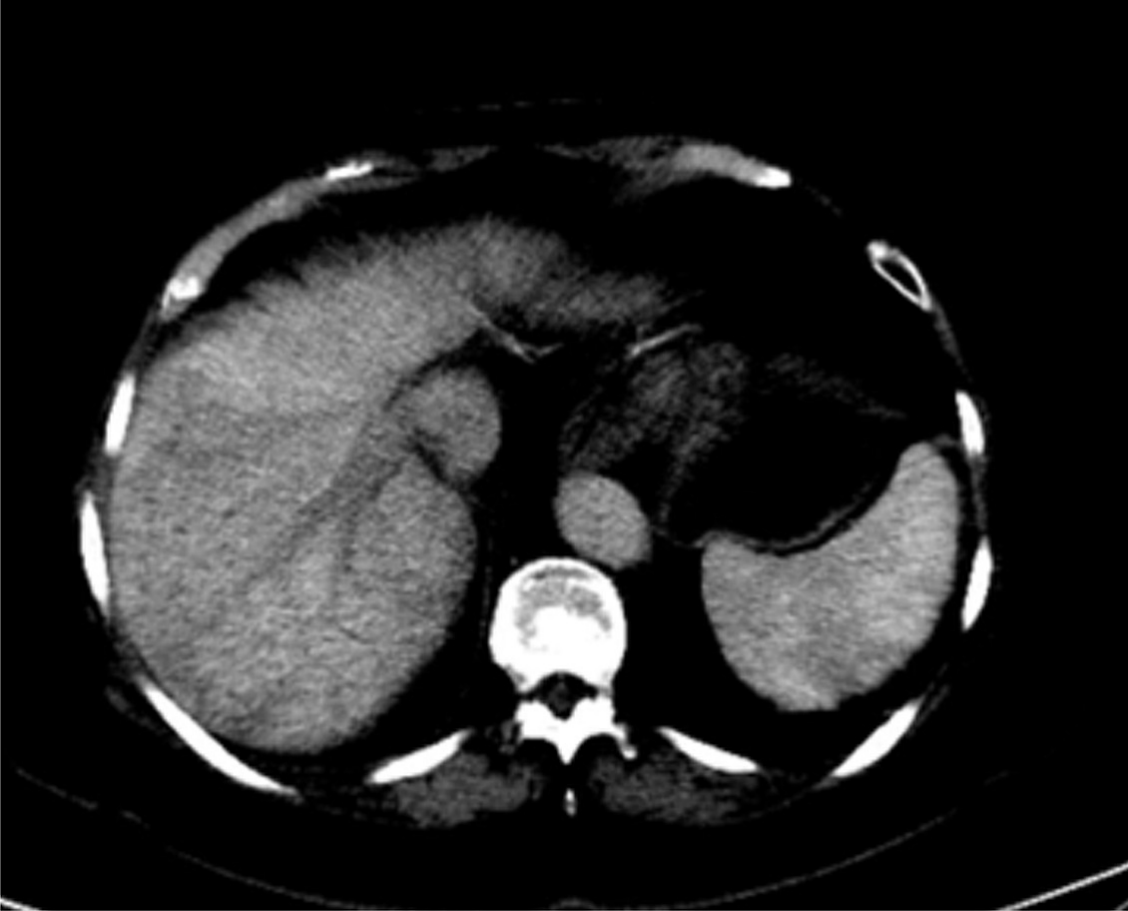
Follow-up scan 6 months after initial CT. No improvement despite anticoagulation.

Owing to the significant cardiovascular comorbidities it was decided that surgical repair was an inappropriate option. The patient was therefore treated conservatively with analgesia and asked to remain on her current anticoagulation therapy. A 6 month follow up CT has not shown a resolution of the hernia or thrombosis, although anticoagulated (Figure 5).

## Discussion

Budd-Chiari syndrome is a rare condition caused by obstruction of the hepatic venous outflow.^1^ Primary Budd-Chiari is diagnosed when the obstruction is due to an intra-venous process of the hepatic veins.^2^ Secondary Budd-Chiari occurs when there is compression or invasion of the hepatic veins.^2^ Signs and symptoms of Budd-Chiari are variable and can include right upper quadrant abdominal pain, abdominal distension from ascites, hepatomegaly, jaundice and acute liver failure.^1^

Hepatic herniation is an extremely rare clinical condition with most cases occurring as a result of congenital diaphragmatic hernias.^3^ Acquired hepatic herniation through diaphragmatic rupture have also been described and are most commonly associated with blunt trauma and incisional herniation.^4^ Abdominal defects with herniation of the liver have been described in 13 reported cases, but only one describes herniation of the right lobe.^3,5–15^ There is also single report of lumbar herniation containing segments of the right hepatic lobe.^16^

Two of the reported cases describing left hepatic lobe herniation also report vascular compromise. Tekin et al.^11^ described portal vein thrombosis in a herniated liver due to liver cirrhosis. Barral et al.^12^ report vascular compromise resulting in a hypodense appearance of the herniated liver on CT. As far as we are aware, no cases of right hepatic lobe herniation with evidence of partial Budd-Chiari syndrome have been described in English literature. In our case we believe the cause of the vascular compromise resulting in partial Budd-Chiari is due to traction from the herniated liver. From the available literature the most common symptoms of liver herniation are abdominal pain, nausea and vomiting. Additionally, in most cases, there are clearly defined hernia edges that are easily palpable on examination. Women also appear to be more likely to suffer with hepatic hernias. In terms of investigation CT scans appear to be highly sensitive in the diagnosis of hepatic herniation. We suggest contrast enhanced CT scan should be undertaken in all patients to assess for any vascular or liver parenchyma compromise. Although hepatic hernias are at very low risk of strangulation due to the wide hernia necks, we have found that Budd-Chiari syndrome can be a clinical consequence of hepatic herniation. Treatment of hepatic hernias requires careful consideration by a multidisciplinary team on a case-by-case basis.

## Conclusions

We present a new case of hepatic herniation, but the first to describe right lobe herniation with signs of partial Budd-Chiari. When cases of liver herniation are suspected, careful evaluation with CT should be performed to exclude potential vascular compromise.

## Acknowledgment

We would like to thank Dr M Williams BM BCh, MA, FRCR, MBA for his initial report on the case and continued support.

## Learning points

1. Hepatic herniation is a rare clinical condition commonly associated with congenital diaphragmatic herniation or acquired through blunt diaphragmatic trauma.
2. Budd-Chiari syndrome can be a consequence of abdominal hepatic herniation.

## Consent

Full written consent has been obtained from the patient for the publication of this case report, including all attached images.
